# Hippocampal subfield volume alterations and associations with severity measures in long COVID and ME/CFS: A 7T MRI study

**DOI:** 10.1371/journal.pone.0316625

**Published:** 2025-01-13

**Authors:** Kiran Thapaliya, Sonya Marshall-Gradisnik, Natalie Eaton-Fitch, Markus Barth, Maira Inderyas, Leighton Barnden

**Affiliations:** 1 National Centre for Neuroimmunology and Emerging Diseases, Griffith University, Australia; 2 School of Information Technology and Electrical Engineering, The University of Queensland, Brisbane, Australia; Jikei University School of Medicine, JAPAN

## Abstract

Long COVID and Myalgic Encephalomyelitis/Chronic Fatigue Syndrome (ME/CFS) patients share similar symptoms including post-exertional malaise, neurocognitive impairment, and memory loss. The neurocognitive impairment in both conditions might be linked to alterations in the hippocampal subfields. Therefore, this study compared alterations in hippocampal subfields of 17 long COVID, 29 ME/CFS patients, and 15 healthy controls (HC). Structural MRI data was acquired with sub-millimeter isotropic resolution on a 7 Telsa MRI scanner and hippocampal subfield volumes were then estimated for each participant using FreeSurfer software. Our study found significantly larger volumes in the left hippocampal subfields of both long COVID and ME/CFS patients compared to HC. These included the left subiculum head (long COVID; p = 0.01, ME/CFS; p = 0.002,), presubiculum head (long COVID; p = 0.004, ME/CFS; p = 0.005), molecular layer hippocampus head (long COVID; p = 0.014, ME/CFS; p = 0.011), and whole hippocampal head (long COVID; p = 0.01, ME/CFS; p = 0.01). Notably, hippocampal subfield volumes were similar between long COVID and ME/CFS patients. Additionally, we found significant associations between hippocampal subfield volumes and severity measures of ‘Pain’, ‘Duration of illness’, ‘Severity of fatigue’, ‘Impaired concentration’, ‘Unrefreshing sleep’, and ‘Physical function’ in both conditions. These findings suggest that hippocampal alterations may contribute to the neurocognitive impairment experienced by long COVID and ME/CFS patients. Furthermore, our study highlights similarities between these two conditions.

## Introduction

Long COVID and Myalgic Encephalomyelitis/Chronic Fatigue Syndrome (ME/CFS) are complex conditions that affect multiple parts of the body. Both illnesses share a range of symptoms including post-exertional malaise, neurocognitive impairment, fatigue, sleep disturbance, pain, and physical disability [1–4].

The COVID-19 pandemic has impactedmillions worldwide, with many individuals experiencing long-term health issues, including effects on the brain [5]. Approximately10% of individuals infected with COVID-19 develop long COVID [6] which is defined as the continuation or development of new symptoms 3 months after the initial infection with the virus, with these symptoms lasting for at least 2 months with no other explanation [7]. Studies suggest that 13–58% of long COVID patients meet ME/CFS criteria [8–10] and exhibit similar symptoms [11, 12]. Both conditions commonly affect brain function, causing problems with concentration [13], decision-making, and information processing [2].

The hippocampus is a vital part of the brain responsible for learning and memory functions [14]. It consists of several subfields, each with specific roles, such as memory performance, memory integration, and delayed recall [15]. Previous studies have reportedreduced volumes of the cornu ammonis, fimbria, subiculum, presubiculum, and parasubiculum in neurodegenerative diseases [16–19]. Conversely, larger volumes of the left subiculum, presubiculum, and fimbria have been observed in ME/CFS patients [20].

Our previous research identified changes in hippocampal subfield volumes in ME/CFS patients using 3T MRI [20]. While long COVID and ME/CFS share similar symptoms, studies have not yet examined hippocampal subfield volumes in long COVID patients. Therefore, this study aims to use ultra-high field 7 Tesla MRI 1) to investigate alterations in hippocampal subfields in long COVID and ME/CFS patients compared to healthy controls (HC), and 2) explore associations between the size of different hippocampal subfields and with the severity measures in both conditions.

## Materials and methods

### Participant recruitment

The study was approved by the Griffith University Human Research Ethics Committee (ID: 2019/1005, 2021/518, 2022/666), conducted in accordance with the relevant guidelines and regulations under the Helsinki Declaration. Written informed consent was obtained from all participants. This cross-sectional investigation was conducted at the National Centre for Neuroimmunology and Emerging Diseases (NCNED) on the Gold Coast, Queensland, Australia. Participants were recruited as described by Thapaliya et al. [21] between 28^th^ July 2021 and 9^th^ August, 2023. Long COVID patients were eligible if their symptoms persisted for at least three months following COVID-19 infection, as defined by the WHO working case definition [7]. ME/CFS patients were recruited if they met the Canadian Consensus Criteria (CCC) and/or International Consensus Criteria (ICC) for diagnosis [2, 22], had received a formal diagnosis of ME/CFS by a physician, and reported no history of COVID-19 infection. Healthy controls were recruited if they reported no chronic health conditions, underlying illness and had no current or prior COVID-19 infection. Participants were aged between 18 and 65 years. Medical histories were reviewed to identify comorbid symptoms or exclusionary diagnoses including mental illness, malignancies, autoimmune, neurological, or cardiovascular diseases. Female participants were excluded if they were pregnant and/or breastfeeding. Ultimately, 17 long COVID as defined by the WHO clinical case definition [7], 30 ME/CFS patients fulfilling the CCC and/or ICC criteria [2, 22], and 15 age-matched HC subjects were included in this study. Table 1 provides demographic information.

**Table 1:** Demographic and clinical characteristics of ME/CFS, long COVID patients, and HC. Superscripts a, b, and c label the p-values for long COVID vs HC, ME/CFS vs HC, and long COVID vs ME/CFS respectively. F = Female, M = male, ICV = intracranial volume.

Diagnostic criteria	ME/CFS (n = 29)	Long COVID (n = 15)	HC (n = 15)	p-value
	17 CCC and ICC 12 CCC only	WHO	N/A	
Age	43.31 ± 11.24	51.65 ± 11.26	38.26 ± 12.74	0.18^a^, 0.004^b^, 0.023^c^,
F/M	23/7	11/4	10/5	N/A
ICV	974,630.76 ±165,764.26	1,085,129.17 ± 262,361.73	990,048.89 ± 132,754.36	0.94^a^, 0.22^b^, 0.16^c^
Duration (years)	13.24±11.13	0.60±0.46	N/A	0.15^c^
Pain	38.4±18.32	52.14±22.1	88.5±17.6	<0.001^a^, <0.001^b^, 0.04^c^
Severity of fatigue	3.8±0.84	3.4 ± 0.5	N/A	0.1^c^
Impaired concentration	3.5±0.93	3±0.75	N/A	0.06^c^
Unrefreshing sleep	3.75±0.78	3.06±1.43	N/A	0.04^c^
Physical function	33.14±24.5	66.3±25.45	97±10.3	<0.001^a^, <0.001^b^, <0.001^c^

#### Symptom severity measures

Symptom severity was assessed using the Research Registry questionnaire developed by NCNED with the Centres for Disease Control and Prevention (CDC) and distributed online through Lime Survey and Redcap. Validated patient-reported outcome measures were used to evaluate participant’s quality of life (QoL) and functional capacity. The 36-item short-form health survey (SF-36) [23] was administered to both long COVID and ME/CFS [24]. SF36 scores were assigned values between 0 and 100, and the average was calculated for each domain.

For subsequent correlation analysis, the severity measures for ‘Duration of illness’, ‘Pain’, and ‘Physical function’ were extracted from SF36v2, while the severity of ‘Fatigue’, ‘Impaired concentration’, and ‘Unrefreshing sleep’ was obtained via the NCNED Research Registry questionnaire. The severity of ‘Pain’ and ‘Physical function’ was assessed on a 0 to 100 point scale, 0 represented very severe symptoms and 100 indicatedno symptoms. The severity of ‘Fatigue’, ‘Impaired concentration’, and ‘Unrefreshing sleep’ was scored on a five-point scale: 1) very mild; 2) mild; 3) moderate; 4) severe; and 5) very severe.

#### MRI scans and data processing

MRI data was acquired using the same MRI protocol as published previously [21]. In brief, MRI was performed on a 7 T whole-body MRI research scanner (Siemens Healthcare, Erlangen, Germany) with a 32-channel head coil (Nova Medical Wilmington, USA). T1-weighted data was acquired using a Magnetisation prepared 2 rapid acquisition gradient echo sequence (MP2RAGE) as in [25]. MP2RAGE data was acquired sagittally using the following parameters: repetition time (TR) = 4300 ms, echo time (TE) = 2.45 ms, inversion times: first (TI1) = 840 ms, second TI2 = 2370 ms, flip angles FA1 = 5°, FA2 = 6° and spatial resolution = 0.75 mm^3^ with matrix size = 256 × 300 × 320.

MP2RAGE data was processed similarly to our previous publications [21]. In brief, MP2RAGE data was anatomically segmented using FreeSurfer version 7.4.1 [26] (https://surfer.nmr.mgh.harvard.edu/) with the default FreeSurfer command ‘recon-all’ on a Macintosh computer (Operating system: Catalina, RAM = 36GB, and core: 8). The ‘recon-all’ processing pipeline includes motion correction, non-linear spatial normalisation to Talairach space, intensity normalization, removal of non-brain tissue, cortical parcellation, sub-cortical segmentation, grey and white matter boundary tessellation, automated topology correction, and surface deformation. Detailed information about the pipeline can be found at (https://surfer.nmr.mgh.harvard.edu/fswiki/recon-all).

**Fig 1:**
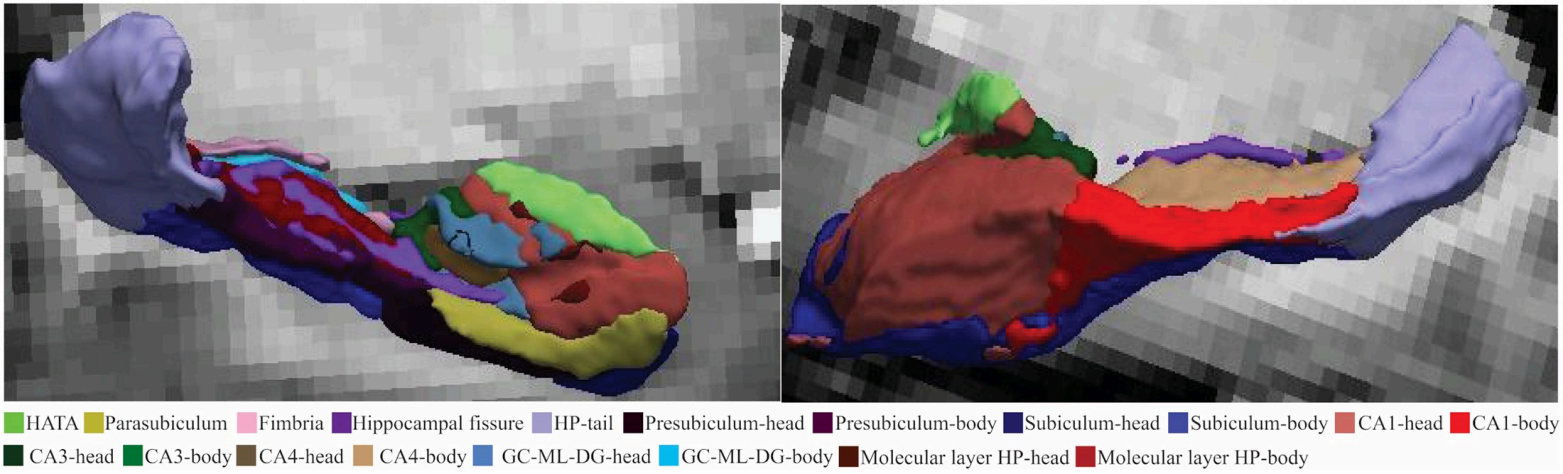
Hippocampal subfield segmentation of a healthy participant overlaid on a reference T1-weighted image. Different colors representdistinct subfields.

Hippocampus subfield volumes were segmented using the FreeSurfer 7.4.1 hippocampus subfield module [27] as shown in Fig 1 similar to our previous publication [20]. Using this module, the left and right hippocampal subfields: hippocampal: head, body, and tail; cornu ammonis (CA1, CA3, and CA4); head and body of subiculum, presubiculum, granular cell layers of the dentate gyrus (GC-ML-DG), molecular layer of the hippocampus (HP); parasubiculum, fissure, fimbria, and hippocampus-amygdala transition area (HATA) were defined. All hippocampal subfields were visuallyinspected to ensure distortion-free segmentation. Two long COVID and one ME/CFS patient were excluded from the analysis due to inadequate segmentation, resulting in a final inclusion of 15 long COVID and 29 ME/CFS patients.

### Statistical analysis

Multivariate general linear model (GLM) statistical analysis was performed to examine hippocampal subfield volume differences among long COVID and ME/CFS patients, relative to HC using SPSS version 29. After confirming homogeneity using Levene’s test, the GLM was applied to assess differences across the three groups. Correction for multiple comparisons was conducted using the Bonferroni method. Then Spearman correlations were performed to investigate the relationships between hippocampal subfield volumes and severity measures in long COVID and ME/CFS patients, also using SPSS version 29. The normality condition for data was assessed prior to the correlations using the Shapiro-Wilk test available in the SPSS version 29. Age, sex, and total intracranial volume were included as nuisance covariates in both analyses. Multiple comparison correction was applied to account for testing across the three groups.

## Results

### Group comparison: Long COVID vs. HC

**Fig 2:**
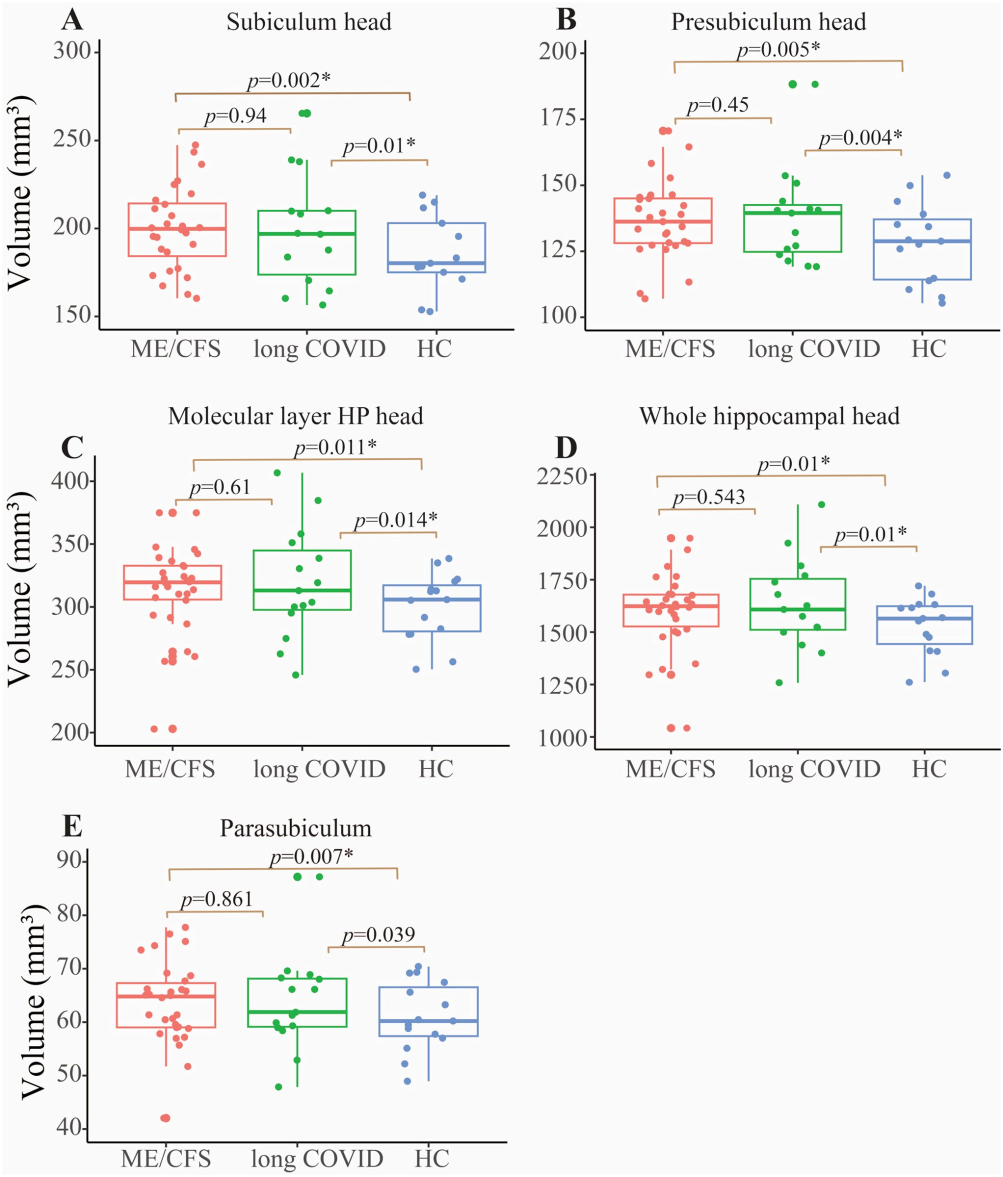
Plots of estimated volumes for five hippocampal subfields. In four cases (A, B, C and D), ME/CFS (red) and long COVID (green) were larger than HC (blue) and were similar for ME/CFS and long COVID. For one case (E), a subfield volume was significantly larger only in ME/CFS (red) compared to HC (blue). A) left subiculum head, B) left presubiculum head, C) molecular layer HP head, and D) whole hippocampal head and E) left parasubiculum. Differences between ME/CFS and long COVID were not significant.

Long COVID patients exhibited significantly *larger* subfield volumes in the left: subiculum head (*p* = 0.01), presubiculum head (*p* = 0.004), molecular layer HP head (*p* = 0.014), and whole hippocampal head (*p* = 0.01) after adjusting for multiple group comparisons (see Fig 2).

### Group comparison: ME/CFS vs. HC

ME/CFS patients also exhibited significantly larger subfield volumes in the left: subiculum head (*p* = **0.002**), pre-subiculum head (*p* = **0.005**), para-subiculum (*p* = **0.007**), molecular layer HP head (*p* = **0.011**), and whole hippocampal head (*p* = **0.01**) after adjusting for multiple group comparisons (Fig 2).

### Group comparison: Long COVID vs. ME/CFS

There were no significant differences in hippocampal subfield volumes between long COVID and ME/CFS patients. Detailed subfield volumes for both conditions can be found in S1 Table.

#### Hippocampal subfield volume correlations with severity measures in long COVID

**Fig 3:**
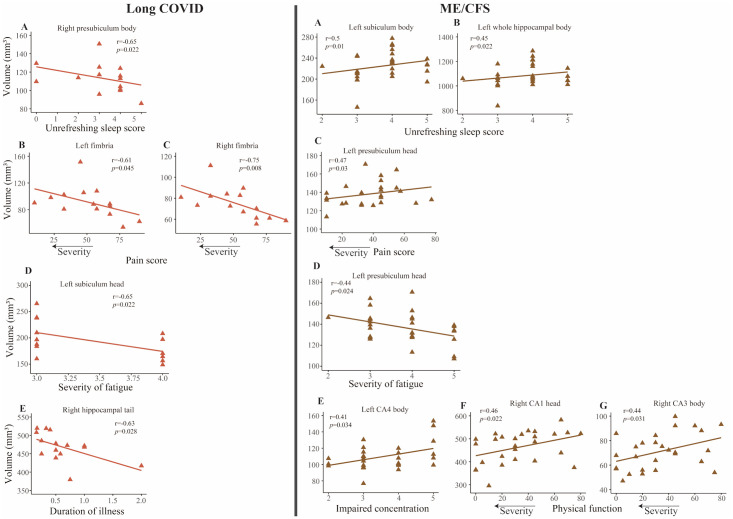
Correlation between hippocampal subfield volumes and clinical measures for long COVID (left) and ME/CFS patients (right). Left: (A) right presubiculum body volume with ‘Unrefreshing sleep’ score, (B, C) bilateral fimbria volumes with a ‘Pain’ score with larger volume associated with more severe pain (lower pain score), (D) left subiculum head with ‘Severity of Fatigue’, and (E) right hippocampal tail with ‘Duration of illness’. Right: (A,B) left subiculum and whole hippocampal body volume correlation with the ‘Unrefreshing sleep’ score, (C) left subiculum and fimbria volumes were correlated with ‘Pain’ score with lower volume associated with more severe pain (lower pain score), (D) left presubiculum head associated with ‘Severity of fatigue’, (E) left CA4 body volume associated with ‘Impaired concentration’, and (F, G) right CA1 head and CA3 body volumes associated with ‘Physical function’ with lower volume associated with lower physical function (lower physical function score). The Y-axis is the volume in mm^3^ and the X-axis is the clinical scores. Lines are the linear regression fit.

In long COVID patients, we observed associations between hippocampal subfield volumes and severity measures of ‘Unrefreshing sleep’, ‘Pain’, ‘Severity of fatigue’, and ‘Duration of illness’ (see Fig 3).

A significant negative correlation was found between Unrefreshing sleep’ and the right presubiculum body volume (r = -0.65, *p* = 0.022). Similarly, ‘Pain’ intensity showed negative correlations with volumes in several left hippocampal subfields including presubiculum body (r = -0.69, *p* = 0.018), fimbria (r = -0.61, *p* = 0.045), HATA (r = -0.67, *p* = 0.023), and the right fimbria (r = -0.75, *p* = 0.008) volume (Fig 3). ‘Severity of fatigue’ exhibited a negative relationship with left subiculum head volume (r = -0.65, *p* = 0.02) while ‘Duration of illness’ was negatively associated with right hippocampal tail volume (r = -0.63, *p* = 0.028) (see Fig 3).

#### Hippocampal subfield volume correlations with severity measures in ME/CFS

In ME/CFS patients, several severity measures, ‘Unrefreshing sleep’, ‘Pain’, ‘Severity of fatigue’, ‘Impaired concentration’, and ‘Physical function’ were associated with hippocampal subfield volumes (see Fig 3).

A significant positive correlation was observed between ‘Unrefreshing sleep’ and left subiculum body (r = 0.5, *p* = 0.01), and whole hippocampal body volume (r = 0.45, *p* = 0.022) (see Fig 3). Similarly, ‘Pain’ and left: presubiculum head (r = 0.47, *p* = 0.03), ‘Impaired concentration’ and left CA4 body volume (r = 0.41, p = 0.034), and ‘Physical function’ and Right: CA1 head (r = 0.46, *p* = 0.022) and CA3 body volumes (r = 0.44, *p* = 0.031) showed significant positive correlations (see Fig 3). Conversely, ‘Severity of fatigue’ exhibited a negative correlation with the left presubiculum head volume (r = -0.44, *p* = 0.024) (see Fig 3).

## Discussion

Our study using ultra-high field 7T MRI found alterations in the hippocampal subfields of both long COVID and ME/CFS patients compared to HC. Notably, we identified larger volumes on the left in: subiculum head, pre-subiculum head, molecular layer HP head, and whole hippocampal head; in both conditions. Furthermore, we found significant associations between hippocampal subfield volumes and severity measures ‘Unrefreshing sleep’, ‘Pain’, ‘Severity of fatigue’, ‘Impaired concentration’, ‘Physical function’, and ‘Duration of illness’ in both conditions. These shared hippocampal subfield volume changes may contribute to the neurocognitive symptoms experienced by individualswith long COVID and ME/CFS.

### Group comparison

This study found larger volumes in specific hippocampal subfields in long COVID and ME/CFS patients compared with HC. The hippocampus is a complex region critical for neurocognitive function [28] and is often affected in neurodegenerative diseases [29]. Importantly, a large proportion of both conditions (70% of long COVID) and (83% of ME/CFS) [30] patients suffer from neurocognitive problems [31].

Recently, larger left subiculum and pre-subiculum head volumes were reported in ME/CFS patients compared to HC [20]. A study of COVID-19 survivors also showed an increase in the grey matter volume in the bilateral hippocampus regions compared to non-covid-19 volunteers [32]. Our study found larger volumes in the left subiculum head, presubiculum head, molecular layer hippocampal head, and whole hippocampal head in long COVID and ME/CFS patients compared with HC. These enlarged volumes may be due to increased neurogenesis and/or functional compensation. Increased hippocampal neurogenesis could be a response to environmental factors and/or stress [33, 34] known to trigger ME/CFS symptoms. An animal study supports this notion, as lower hippocampal neurogenesis was observed in poor-learning rats compared to better-learning rats [35]. Sex hormones also influence neurogenesis within the hippocampus. Estrogens modulate neurogenesis in females [36], and androgens play a similar role in males [37]. Both have been reported to be higher in ME/CFS than HC [38]. Addtionally, there is a strong connection between the hippocampus and brainstem regions [39]. The hippocampus may undergo hypertrophy to compensate for brainstem deficits, which have been reported in both long COVID and ME/CFS patients [21, 40, 41].

The similar hippocampal subfield volumes observed in both long COVID and ME/CFS patients align with the known overlap of symptoms between these conditions [4]. These findings are consistent with our previous work demonstrating similar brainstem volumes in different long COVID and ME/CFS cohorts [21]. Additionally, dysfunction of Transient receptor potential cation channel subfamily M member 3 (TRPM3) receptors which are highly expressed in the central nervous system [42], has been linked to both long COVID and ME/CFS [43]. Given that neurocognitive impairment is a shared symptom of both conditions [4], our findings suggest that structural alterations within the hippocampus might contribute to the neurocognitive dysfunction seen in both long COVID and ME/CFS.

### Correlation with severity measures in long COVID and ME/CFS patients

#### Severity of unrefreshing sleep

Most long COVID and ME/CFS patients experience unrefreshing sleep and sleep deprivation [44]. In our study of long COVID patients, the right pre-subiculum body showed a negative correlation with the ‘Severity of unrefreshing sleep’ (r = -0.65, *p* = 0.02), indicating that reduced sleep quality was associated with smaller hippocampal subfield volume. In contrast, in ME/CFS patients, we found a positive correlation between ‘Severity of unrefreshing sleep’ and the contralateral left subiculum body volume (r = 0.5, *p* = 0.01) and whole hippocampal body volumes (r = 0.45, *p* = 0.022), indicating that larger volumes were associated with greater sleep problems. Our previous research in ME/CFS patients also showed an association between greater sleep disturbance and larger hippocampal subfield volumes [20]. A recent longitudinal study [45] showed that patients with post-COVID ME/CFS suffered from more severe sleep disturbance compared to those with post-COVID condition. The contrasting correlations for long COVID and ME/CFS patients could relate to their different symptom severity.

#### Pain

In long COVID patients, we observed a negative correlation between ‘Pain’ and volumes of the left presubiculum body (r = -0.69, *p* = 0.018), left fimbria (r = -0.61, *p* = 0.044), left HATA (r = -0.67, *p* = 0.023) and right fimbria (r = -0.75, *p* = 0.008) (see Fig 3). In contrast, in ME/CFS, we observed a positive correlation between ‘Pain’ and left presubiculum head volume (r = 0.47, *p* = 0.03), indicating that lower volume is associated with increased ‘Pain’ intensity (see Fig 3). Our previous study [20] also showed a positive correlation between ‘Pain’ and hippocampal subfield volumes in a different cohort of ME/CFS. Similarly, a study in elderly women found that enlarged hippocampal subfield volumes were associated with ‘Pain’ intensity [46], and Smallwood et al. [47] reported increased grey matter volume in the hippocampus of chronic pain patients. The association between hippocampal subfield volumes and ‘Pain’ intensity could be explained by impaired cortico-limbic connectivity, which integrates pain characteristics into the hippocampus [48]. These findings demonstrate a link between pain intensity and altered hippocampal subfields in both long COVID and ME/CFS. A recent longitudinal study [45] showed that pain scores were more affected in Post COVID Syndrome-ME/CFS patients. However, while Post COVID Syndrome patients showed improvement in the pain score in the follow-up there was only a minor improvement among ME/CFS patients. The contrasting correlations for long COVID and ME/CFS patients could be due to the permanency of symptoms in ME/CFS, whereas these symptoms might improve over time in long COVID patients [45].

#### Severity of fatigue

Fatigue is one of the most common symptoms experienced by long COVID and ME/CFS patients [4]. Our study found a negative correlation between the ‘Severity of fatigue’ and hippocampal subfield volumes in both conditions. In long COVID, this correlation was observed in the left subiculum head volume (r = -0.65, *p* = 0.02), while in ME/CFS patients, it was in the left presubiculum head (r = -0.44, *p* = 0.024). This suggests that greater fatigue severity is associated with smaller hippocampal subfield volumes in both conditions. These findings align with other research. A recent study by Wasson et al [49] found an association between reduced hippocampal subfield volume and fatigue. Our previous work [20] similarly showed a negative association between fatigue severity and hippocampal subfield volumes in ME/CFS patients.

#### Duration of illness

In long COVID patients, we observed a negative correlation between the ‘Duration of illness’ and right hippocampal tail volume (r = -0.63, *p* = 0.028), indicating that this smaller hippocampal subfield volume is associated with a longer illness duration. A study in long COVID patients reported severity of cognitive impairment increased with illness duration [13]. Interestingly, we did not find a significant correlation between the ‘Duration of illness’ and hippocampal subfield volumes in ME/CFS patients. This difference could be due to the disease course. Altered Hippocampal volumes in ME/CFS may be progressive only in the early stages of the disease. Furthermore, the average illness duration in our study was markedly shorter for long COVID patients (0.60±0.46 years) compared to ME/CFS patients (13.24±11.13).

#### Impaired concentration

ME/CFS patients showed a positive correlation between severity of ‘Impaired concentration’ and left CA4 body volume (r = 0.41, *p* = 0.034), indicating that a larger volume is associated with greater impairment in concentration. This supports our recent report of a positive correlation between hippocampal subfield volumes and ‘Impaired concentration’ in a different cohort of ME/CFS patients [20]. Another ME/CFS study using diffusion tensor imaging showed abnormal regression with ‘Impaired concentration’ in the hippocampus region [50]. Lim et al. [51] demonstrated an association between hippocampal subfield volumes and constructional recall scores and memory tests. Similarly, a larger cornu ammonis volume was associated with complex figure delayed recall [52], indicating that the CA4 subfield plays a crucial role in memory retrieval.

#### Physical activity

ME/CFS patients often experience limited physical activity due to post-exertional malaise [53]. We found a positive correlation between ‘Physical activity’ levels and hippocampal subfield volumes on the right: CA1 head (r = 0.46, p = 0.022) and CA3 body (r = 0.44, p = 0.031) in ME/CFS patients, indicating that smaller subfield volumes are associated with lower ‘Physical activity’. A Brain-derived neurotrophic factor (BDNF) is elevated by physical activity in the hippocampus and is crucial for neurogenesis and neuroplasticity [54], which is impaired in ME/CFS patients [54]. Interestingly, no such relationship between physical activity and hippocampal subfield volumes was observed in long COVID patients. This difference could be due to the potentially greater disease severity and longer illness duration in ME/CFS patients compared to long COVID patients.

#### Contrasting clinical associations in long COVID and ME/CFS

We observed some contrasting relationships between between hippocampal subfield volumes and clinical measures in long COVID and ME/CFS. Hippocampal subfield volumes were negatively correlated with unrefreshing sleep and pain scores in long COVID patients, whereas the pain score correlation was positive in ME/CFS patients. This difference might be due to symptom severity. Most ME/CFS patients met the stringent diagnostic criteria of CCC or ICC, unlike many long COVID patients.

Previously, we demonstrated that ME/CFS patients meeting stringent criteria (CCC or ICC) exhibited altered hippocampal subfield volumes compared to those meeting less stringent criteria (Fukuda) [20]. Similarly, another study using diffusion tensor imaging revealed tissue microstructural changes in ME/CFS patients meeting CCC or ICC criteria but not in those meeting only the Fukuda criteria [50]. A recent longitudinal study [45] indicated that sleep disturbances and pain scores were more significantly affected in Post COVIDSyndrome-ME/CFS patients, and while Post COVID Syndrome patients showed improvement in pain scores over time, improvement was minor among ME/CFS patients.

The contrasting relationships between hippocampal subfield volumes and clinical measures in long COVID and ME/CFS patients could be attributed to the more severe symptoms of ME/CFS. Further longitudinal studies are needed to determine whether long COVID patients who eventually meet ME/CFS criteria exhibit similar correlations between hippocampal subfield volumes and clinical measures.

### Limitations

Hippocampal subfield volumes are known to differ with sex and handedness (left versus right) [55, 56]. However, due to our limited sample size, this study was unable to explore these sex and handedness differences in long COVID and ME/CFS patients. Another limitation is that this study design was cross-sectional. Therefore, a longitudinal study is needed to confirm whether the hippocampal volume changes are progressive in long COVID and ME/CFS patients. A longitudinal study may potentially confirm whether similar volume behaviour persists in long COVID and ME/CFS patients. Additionally, all clinical measures for long COVID and ME/CFS were obtained via online surveys, which may yield under and over-reporting of clinical measures.

## Conclusion

Our analysis revealed altered hippocampal subfield volumes in both long COVID and ME/CFS patients. Notably, no significant differences in hippocampal subfield volumes were observed between two conditions. Furthermore, we found significant associations between hippocampal subfield volumes and severity measures in both long COVID and ME/CFS patients. These findings suggest that structural alterations in the hippocampus may contribute to overlapping symptoms, such as cognitive problems in long COVID and ME/CFS patients. Future research investigating long COVID and ME/CFS patients together could provide deeper insights into the potential neurological underpinnings shared by these conditions.

## Supporting information

S1 Table The mean and standard deviation hippocampal subfield volumes for ME/CFS, long COVID patients and healthy controls (HC). (DOCX)
