# Supplementary material for: Hippocampal subfield volume alterations and associations with severity measures in long COVID and ME/CFS: A 7T MRI study
Source: PLoS One. 2025 Jan 13;20(1):e0316625. doi: 10.1371/journal.pone.0316625 (PMC11729965; doi:10.1371/journal.pone.0316625)
Supplement: S1 Table — (DOCX) [file pone.0316625.s001.docx]

Table S1 shows the mean and standard deviation hippocampal subfield volumes for ME/CFS, long COVID patients and healthy controls (HC).

|  | Volume in mm^3^ | | |
| --- | --- | --- | --- |
|  | ME/CFS | long COVID | HC |
| Left Hippocampal | | | |
| Whole | 3213.1 ±231.85 | 3224.6 ±374.21 | 3117.5 ±224.52 |
| Head | 1618.4 ±148.54 | 1632.5 ±216.37 | 1528.8 ±134.71 |
| Body | 1092.1 ±76.90 | 1075.8 ±118.66 | 1067.9 ±79.70 |
| Tail | 502.6 ±62.23 | 516.3 ±77.04 | 520.8 ±50.97 |
| CA1-head | 482.1 ±45.99 | 492.0 ±64.32 | 461.3± 46.56 |
| CA1-body | 104.8 ±12.40 | 107.7 ±14.55 | 109.8± 20.01 |
| CA3-head | 107.3 ±12.69 | 106.7 ±17.11 | 102.1± 10.97 |
| CA3-body | 74.8 ±13.06 | 77.7 ±12.23 | 75.9 ±12.11 |
| CA4-head | 115.9 ±12.27 | 117.7 ±16.20 | 110.8± 10.72 |
| CA4-body | 110.7 ±14.32 | 109.4 ±12.35 | 109.4 ±12.01 |
| Subiculum-head | 198.0 ±24.89 | 195.8 ±33.71 | 180.7 ±24.37 |
| Subiculum-body | 228.5 ±21.69 | 215.8 ±36.14 | 215.5± 26.61 |
| Presubiculum-head | 136.6 ±14.64 | 137.8 ±17.89 | 128.0 ±15.19 |
| Presubiculum-body | 153.2 ±23.84 | 148.7 ± 16.40 | 143.8 ±22.19 |
| Parasubiculum | 64.3 ±6.63 | 63.7 ±8.97 | 61.0 ±6.46 |
| Molecular layer-head | 317.8 ±28.53 | 319.1 ±43.98 | 300.3 ±26.55 |
| Molecular layer- body | 204.7 ±15.82 | 203.6 ±24.23 | 201.1 ±17.65 |
| GC-ML-DG-head | 141.2 ±14.50 | 142.2 ±20.62 | 133.7 ±12.44 |
| GC-ML-DG-body | 123.4 ±13.35 | 122.9 ±13.04 | 122.4 ±11.69 |
| Fimbria | 92.0 ± 17.56 | 90.1 ± 22.59 | 90.0± 16.38 |
| Fissure | 166.4 ±25.60 | 170.7 ±25.44 | 171.4 ±19.49 |
| HATA | 55.2 ±8.22 | 57.5 ± 13.13 | 50.9± 5.64 |
| Right | | | |
| Whole | 2846.6 ±409.18 | 2867.5 ±342.75 | 2780.7 ±376.34 |
| Head | 1472.3 ±232.46 | 1486.9 ±241.05 | 1404.1 ±196.63 |
| Body | 925.4 ±122.28 | 911.9 ±102.37 | 913.8 ±133.00 |
| Tail | 449.0 ±81.07 | 468.7 ±39.18 | 462.8 ±73.30 |
| CA1-head | 462.7 ±66.73 | 484.4 ±82.48 | 442.0 ±60.18 |
| CA1-body | 98.8 ±17.00 | 102.4 ±19.22 | 100.6 ±19.43 |
| CA3-head | 95.4 20.61 | 92.5 21.91 | 89.0 ±17.68 |
| CA3-body | 70.1 ±14.36 | 69.9 ±15.25 | 68.1 ±16.15 |
| CA4-head | 103.1 ±20.31 | 101.5 ±17.18 | 98.4 ±16.27 |
| CA4-body | 96.3 ±15.10 | 95.9 ±15.45 | 95.5 ±17.45 |
| Subiculum-head | 173.2 ±29.15 | 172.8 ±29.46 | 166.8 ±22.22 |
| Subiculum-body | 183.7 ±28.01 | 174.1 ±20.26 | 177.1 ±28.88 |
| Presubiculum-head | 117.4 ±22.13 | 114.4 ±16.87 | 111.2 ±18.17 |
| Presubiculum-body | 118.1 ±21.12 | 113.5 ±15.66 | 115.5 ±16.36 |
| Parasubiculum | 51.9 ± 10.58 | 51.7 ± 9.65 | 49.9 ±9.91 |
| Molecular layer-head | 293.5 ±44.85 | 296.8 ±46.03 | 281.3 ±37.78 |
| Molecular layer- body | 178.7 ±26.18 | 175.7 ±22.74 | 176.0 ±28.93 |
| GC-ML-DG-head | 126.0 ±23.59 | 124.3 ±21.28 | 120.2 ±19.16 |
| GC-ML-DG-body | 106.2 ±16.10 | 106.1 ±14.58 | 106.6 ±17.79 |
| Fimbria | 73.5 ± 11.89 | 74.3 ± 14.58 | 74.4 ±9.05 |
| Fissure | 141.1 ±29.07 | 145.0 ±31.27 | 139.5 ±21.03 |
| HATA | 49.0 ± 11.23 | 48.6 ± 12.28 | 45.3 ±11.19 |
